# The need of the hour – To make periodontics more interesting for undergraduate students!

**DOI:** 10.4103/0972-124X.70826

**Published:** 2010

**Authors:** Chini Doraswamy Dwarakanath

**Affiliations:** *President, Department of Periodontology, Director of Post Graduate Studies, Oxford Dental College and Hospital, Hosur Road, Bangalore-560 068, India. E-mail: drdwarakcd@yahoo.co.in*

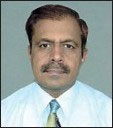



The Dental Council of India (DCI), in its revised curriculum for Bachelor of Dental Surgery (BDS), has extended the course to 5 years, while at the same time eliminating the compulsory rotatory internship. During this process some of the subjects were jiggled and redistributed. Periodontics, to the much consternation of many, was placed in the 4th year along with orthodontics, oral medicine and pedodontics, where as prosthodontics, conservative dentistry, oral surgery and community dentistry were grouped for the 5th and final year. While I do not subscribe to the view expressed by many teachers that the subject of periodontics has been relegated, I certainly feel that in the context of current scenario of dentistry, particularly multidisciplinary aspects, our discipline should have been placed in the 5th year along with restorative dentistry.

Many postgraduate students and young teachers have urged the ISP to represent to the DCI regarding this matter.

Before we actually go to the DCI we need to do little introspection about our method of presenting and teaching the subject to the BDS students. It is unfortunate and painfully true that most of the undergraduate (UG) students consider periodontics singularly uninteresting, the clinical postings to be somehow done with. Other than manual scaling, they do not get to do anything in most of the dental colleges in both their 3rd and 4th years. Hardly are they given cases of advanced and aggressive forms of periodontitis or various gingival enlargements. This is particularly so in those colleges with MDS courses where the postgraduate students take away all the cases with notable findings. Further, they do not get any opportunity either to observe or assist surgical procedures. In other words, they have no idea about the immensely beneficial effects of periodontal therapy. Other than one or two lectures, they hardly get exposure to the interdisciplinary aspects stressing the importance of sound periodontal health before other dental treatment can be taken up.

I strongly advocate and urge all my fellow teachers to institute such measures in UG training which makes periodontics more interesting and attractive to the UG students. Instead of merely going by the quota of work prescribed by the regulatory bodies, if we introduce problem-based learning and training, I am sure it would go a long way to capture the interest of students.

Further it is high time we allow our students, at least during the last part of their clinical postings, to use ultrasonic scalers. Now that comprehensive dentistry has been introduced in the 5th and last year of BDS, these postings should be made meaningful and useful so that students adopt a holistic approach rather than looking at various dental treatments as separate entities. Don’t you think our mind set needs a change?

